# Outcomes and survival trends following pelvic exenteration for locally advanced and recurrent rectal cancer: a 20-Year analysis from a tertiary cancer center in India

**DOI:** 10.1186/s12957-025-04053-0

**Published:** 2025-11-28

**Authors:** M.D Ray, Amit Kumar, Rohan Kapoor

**Affiliations:** Department of Surgical Oncology, All India Institute of Medical Sciences, New Delhi, India

**Keywords:** Pelvic exenteration, Locally advanced rectal cancer, Locally recurrent rectal cancer, R0 resection, Overall survival

## Abstract

**Background:**

Pelvic exenteration (PE) offers a potential cure for selected patients with Locally Advanced Rectal Cancer (LARC) or Locally Recurrent Rectal Cancer (LRRC) invading adjacent pelvic organs. Despite advances in surgical technique and perioperative care, PE remains associated with significant morbidity. This study evaluates long-term oncologic outcomes of PE over 20 years at a high-volume tertiary cancer center in India.

**Methods:**

We retrospectively analysed 97 patients who underwent PE between January 2000 and December 2020. Patients included those with LARC or LRRC, where R0 resection was deemed feasible. Surgical procedures were classified as total pelvic exenteration (TPE) or modified pelvic exenteration (MPE). Data on demographics, operative parameters, pathological features, recurrence pattern and survival were analysed.

**Results:**

Among the 97 patients (median age 59; 80.4% male), 67% had LARC and 33% LRRC. R0 resection was achieved in 71.1%. TPE was more common in LRRC, while MPE predominated in LARC (*p* = 0.014). Common complications included pelvic collection (25.8%) and wound infection (15.5%). The 5-year OS was higher in R0 resection patients (51.9% vs. 12.9%; *p* = 0.013) and those with LARC vs. LRRC (57.0% vs. 10.6%; *p* = 0.032). LRRC had higher recurrences post R0 resection. In the multivariate analysis, the only independent predictors of OS were the initial presentation of the disease and R0 resection.

**Conclusion:**

PE remains a curative strategy for LARC and LRRC following an R0 resection. LRRC is associated with higher recurrence and poorer survival. Optimal outcomes require multidisciplinary evaluation, margin-negative resection, and tailored surgical approaches. This study provides data from a low- and middle-income country setting, where such literature remains limited.

## Introduction

 Rectal carcinoma remains a significant global health burden, ranking as the third most commonly diagnosed cancer and the second leading cause of cancer-related mortality worldwide [1]. A subset of patients, particularly those with locally advanced rectal cancer (LARC), present with direct tumor invasion into adjacent pelvic structures, classified as cT4b disease, posing formidable challenges to surgical resectability and oncological clearance [2]. In approximately 10%–20% of rectal cancer cases, the tumor penetrates adjacent organs or structures without distant metastasis [3, 4].

In such cases, achieving an R0 resection, which is fundamental to curative treatment, requires en bloc multivisceral resection, frequently in the form of pelvic exenteration (PE) [5]. First introduced by Brunschwig in 1948 for palliation in cervical cancer, PE has evolved into a potentially curative intervention for select patients with LARC or locally recurrent rectal cancer (LRRC) [6, 7]. While this procedure offers the potential for long-term survival, it is associated with substantial morbidity, prolonged recovery, and requires complex perioperative and multidisciplinary planning.

The advancement of total mesorectal excision (TME), alongside the integration of multimodal treatment strategies such as neoadjuvant chemoradiotherapy (CRT) or total neoadjuvant therapy (TNT), has markedly improved rectal cancer outcomes by decreasing local recurrence rates and enhancing resectability [8, 9]. Specifically, TNT, which integrates systemic chemotherapy with preoperative radiation, is increasingly preferred for downstaging and enhancing complete pathologic response rates prior to major surgery [10].

PE may be categorized into total, anterior, or posterior, and further subdivided based on levator ani involvement (supralevator vs. infralevator) [11]. Total pelvic exenteration (TPE) involves the removal of the rectum, bladder, internal reproductive organs, distal ureters, and surrounding soft tissue and lymphatics. Anterior exenteration entails the removal of the bladder and reproductive organs while preserving the rectum, typically used in gynecological or urological cancers confined to the anterior compartment. In posterior pelvic exenteration, the resected organs include the posterior vaginal wall, uterus, cervix, and sometimes adnexa in females, while in males, it may involve the seminal vesicles and posterior prostate.

Modified pelvic exenteration (MPE) is a less extensive alternative to TPE, performed for locally advanced or recurrent pelvic malignancies. It involves multivisceral resection with organ preservation when feasible, aiming for R0 resection while minimizing morbidity and preserving urinary or anorectal function.

Advances in surgical technique, patient selection, and perioperative care have led to five-year overall survival rates ranging from 30% to 60% in high-volume centers, even for recurrent cases [12, 13]. However, despite progress, morbidity rates remain high, up to 60%–70%, with complications such as pelvic abscess, wound infection, urinary leaks, and anastomotic failures being common [14]. Hence, contemporary guidelines emphasize the importance of careful patient selection, optimal use of neoadjuvant therapies, and surgical expertise.

This study highlights our institutional experience with pelvic exenteration for locally advanced and recurrent rectal cancers. We aim to analyse both short-term and long-term outcomes in our cohort, focusing on overall survival (OS) and recurrence patterns.

## Methods

### Study design and patient selection

This retrospective observational study included 97 patients who underwent PE for LARC or LRRC between January 2000 and December 2020 at the Department of Surgical Oncology, a high-volume tertiary cancer referral center in India that serves patients from multiple states. The cohort comprised both referred cases, often with recurrent disease or incomplete prior surgeries following neoadjuvant therapy, and in-house cases, diagnosed and managed primarily within our institution, particularly those with LARC deemed suitable for exenteration. Clinical data were retrieved from a prospectively maintained institutional database, capturing demographic characteristics, clinical presentation, prior treatment details, staging, recurrence patterns, and survival outcomes. As anonymized patient records were analyzed without direct patient contact or additional intervention, Institutional Ethics Committee (IEC) approval was not mandated under institutional policy and national ethical guidelines. However, written informed consent was obtained from all patients before surgery, following detailed preoperative counselling regarding the diagnosis, planned procedure, potential risks, and alternative management options, provided in a language they could understand, ensuring voluntary participation and respect for patient autonomy.

#### Inclusion criteria

Eligible patients included those with locally advanced or recurrent rectal cancer with direct invasion or adherence to adjacent pelvic organs or structures, in whom a complete (R0) resection was deemed feasible. Patients were selected with curative intent following a multidisciplinary team (MDT) consensus based on comprehensive clinical and radiological evaluation.

#### Exclusion criteria

Patients were excluded if they had unresectable disease, including major vessel encasement without reconstruction options, lateral pelvic side wall involvement, bony involvement (sacrum/coccyx), peritoneal carcinomatosis, or distant metastases. Other exclusion criteria included poor performance status (ECOG ≥ 3 or Karnofsky < 60), significant comorbidities (severe cardiopulmonary, hepatic, or renal dysfunction), inadequate psychosocial support, or refusal of surgery after counselling. Specifically, patients requiring en bloc sacrectomy or coccygectomy (*n* = 8) were excluded, as these cases represent a distinct surgical subgroup with different complexity, reconstruction requirements, and outcomes. Consequently, this study focuses on outcomes in patients undergoing standard pelvic exenteration without bony resections.

### Preoperative evaluation

All patients underwent a standardized preoperative workup, which included a physical examination, serum carcinoembryonic antigen (CEA) testing, colonoscopy with biopsy for histological confirmation, and contrast-enhanced computed tomography (CT) of the chest and abdomen to rule out distant metastasis. Pelvic magnetic resonance imaging (MRI) was used for local staging and to assess resectability. Positron emission tomography-computed tomography (PET-CT) was performed selectively, with indications including suspected distant metastasis requiring characterization, equivocal findings on CT or MRI needing metabolic assessment, differentiation of post-treatment fibrosis from active recurrence, and preoperative staging in previously operated patients with inadequate prior imaging records.

### Multidisciplinary assessment

At our institution, patients considered for exenterative surgery were evaluated in a MDT meeting, comprising surgical oncologists, radiologists, medical oncologists and radiation oncologists. The MDT assessed: clinical status, radiological resectability by MRI/PET-CT, neoadjuvant therapy response, pelvic organ involvement, absence of unresectable metastases, and the patient’s comorbidities. Only patients with resectable disease, potential for R0 resection, and fitness for major surgery were selected. Patient preferences and informed consent were integral to the decision. In patients where negative margins appeared threatened, neoadjuvant therapy was considered based on tumor location and prior treatment history.

### Surgical techniques: TPE and MPE

All patients underwent pelvic exenteration, classified as either Total Pelvic Exenteration (TPE) or Modified Pelvic Exenteration (MPE), with the approach tailored to tumor extent and anatomical involvement.

#### Total pelvic exenteration (TPE)

TPE involved en bloc resection of the distal colon, rectum, anus, urinary bladder, distal ureters, and internal reproductive organs (uterus, ovaries, fallopian tubes, and occasionally the vagina). This was indicated when tumors involved both anterior and posterior compartments. The procedure necessitated permanent colostomy and urinary diversion, most commonly via an ileal conduit (Bricker technique).

#### Modified pelvic exenteration (MPE)

MPE was performed in selected patients to preserve function where feasible. This included rectal preservation or reconstruction via low anterior resection (LAR) with coloanal or colorectal anastomosis if the distal rectum was not involved. The urinary tract was managed with total cystectomy and diversion similar to TPE. The approach aimed to balance oncologic radicality with functional outcomes based on tumor location and invasion.

MPE involves tailored resections based on disease extent and potential organ preservation. Organ-preserving approaches included bladder preservation in posterior exenteration cases, vaginal preservation when disease spared the anterior wall, and urethral/anal sphincter preservation in selected low-volume central recurrences. However, detailed documentation of these modifications was limited during the study period, preventing analysis of their impact on margins and functional outcomes.

##### Reconstruction of defect

In our cohort, reconstruction was part of the multidisciplinary surgical plan. Primary closure was attempted where feasible. For large perineal or pelvic floor defects, myocutaneous flaps—predominantly VRAM flap—were used to obliterate dead space and ensure wound coverage. Plastic surgeons were involved in these cases.

### Follow-up and surveillance

Patients were followed up at 3–6 month intervals in the outpatient setting. Follow-up assessments included clinical examination and digital rectal exam, serum CEA levels, and routine blood tests such as complete blood count and liver function tests. Imaging with contrast-enhanced CT of the chest, abdomen, and pelvis was performed when clinical or biochemical findings suggested recurrence. PET-CT was used in selected cases for further evaluation. Recurrence was confirmed either histopathologically through image-guided biopsy or cytology or radiologically in the presence of progressive disease. Recurrences were categorized as local (within the pelvic cavity) or distant (outside the pelvis).

### Statistical analysis

All data were analyzed using SPSS v27.0 (IBM Corp., Armonk, NY). Continuous variables were summarized as mean ± standard deviation or median with range, while categorical variables were reported as frequencies and percentages. Group comparisons were made using the Chi-square or Fisher’s exact test for categorical variables and the independent t-test for continuous variables. Survival outcomes were analyzed using the Kaplan–Meier method, and differences between groups were assessed with the log-rank test. Cox proportional hazards regression was used for univariate and multivariate analyses of prognostic factors affecting 5-year overall survival (OS). Hazard ratios (HRs) with 95% confidence intervals (CIs) were reported. A p-value of < 0.05 was considered statistically significant.

## Results

A total of 97 patients with a median age of 59 years (range: 38–84) were included. The majority were male (80.4%). Locally advanced rectal cancer (LARC) accounted for 67% and locally recurrent rectal cancer (LRRC) for 33%. Modified pelvic exenteration (MPE) was performed in 55.7% and total pelvic exenteration (TPE) in 44.3%. R0 resection was achieved in 71.1%. Preoperative chemoradiotherapy or prior radiotherapy was administered in 62.9%, and adjuvant chemotherapy in 77.3%. Baseline patient characteristics and operative details are outlined in Table 1. VRAM flap was done in 11 cases as a part of the reconstruction of the defect.

**Table 1:** Patient characteristics and operative details

Variable	LARC (*n* = 65)	LRRC (*n* = 32)	Total (*n* = 97)	*p*-value (if Significant)
Age, median (range)	58 (38–80)	61 (42–84)	59 (38–84)	–
Sex				
Male	54 (83.1%)	24 (75.0%)	78 (80.4%)	–
Female	11 (16.9%)	11 (16.9%)	19 (19.6%)	–
Tumor site				
Middle/lower rectum	37 (56.9%)	28 (87.5%)	65 (67.0%)	0.047
Upper rectum/Rectosigmoid Junction	28 (43.1%)	4 (12.5%)	32 (33.0%)	-
CEA levels				
< 5 ng/mL	17 (26.2%)	21 (65.6%)	48 (73.8%)	0.013
≥ 5 ng/mL	48 (73.8%)	11 (34.4%)	11 (34.4%)	-
Type of procedure				
Total Pelvic Exenteration (TPE)	20 (30.8%)	23 (71.9%)	43 (44.3%)	0.014
Modified Pelvic Exenteration (MPE)	45 (69.2%)	9 (28.1%)	54 (55.7%)	-
Extent of resection				
R0	54 (83.1%)	15 (46.9%)	69 (71.1%)	0.039
R1/R2	11 (16.9%)	17 (53.1%)	28 (28.9%)	
Preoperative CRT or prior RT	39 (60.0%)	22 (68.8%)	61 (62.9%)	–
Adjuvant chemotherapy	49 (75.4%)	26 (81.3%)	75 (77.3%)	–
Duration of surgery (minutes)	360.0 ± 40	480.7 ± 83.1	–	0.002
Hospital stay (days)	11 ± 6.0	13.4 ± 9.0	–	0.011
Blood transfusion (units)	2.8 ± 1.4	4.3 ± 2.1	–	-

In the analysis of pathologic outcomes(Table 2), no statistically significant differences were observed between the LARC and LRRC groups across all parameters. Most tumors were well or moderately differentiated (90.8% vs. 81.3%), with pT4 status predominating in both groups (87.7% vs. 93.8%). Nodal positivity was similar (60.0% vs. 59.4%, *p* = 0.894). Lymphovascular invasion was more frequent in LRRC (93.7% vs. 78.5%), with a p value of 0.373. whereas Perineural invasion rates were comparable (53.1% vs. 52.3%, *p* = 0.941).

**Table 2:** Pathologic outcomes and postoperative complications

Variable	LARC (*n* = 65)	LRRC (*n* = 32)	*p*-value
Tumor differentiation			
Well Differentiated/Moderately Differentiated	59 (90.8%)	26 (81.3%)	0.630
Poorly Differentiated/mucinous	6 (9.2%)	6 (18.7%)	
Pathologic T stage			
pT4	57 (87.7%)	30 (93.8%)	0.623
pT3	8 (12.3%)	2 (6.2%)	
Pathologic N stage			
pN0	26 (40.0%)	13 (40.6%)	0.894
pN+	39 (60.0%)	19 (59.4%)	
Lymphovascular invasion			
Present	51 (78.5%)	30 (93.7%)	0.373
Absent	14 (21.5%)	2 (6.3%)	
Perineural invasion			
Present	34 (52.3%)	17 (53.1%)	0.941
Absent	31 (47.7%)	15 (46.9%)	

Postoperative morbidity was assessed using the Clavien-Dindo classification. Complications occurred in 48 patients (49.5%), with the majority being Grade II or lower. The most common complication was pelvic collection, observed in 25 patients (25.8%), of which 16 were managed conservatively with antibiotics (Grade II), and 9 required image-guided drainage (Grade IIIa). Wound infection occurred in 15 patients (15.5%), primarily managed with dressing and antibiotics (Grade I–II). Paralytic ileus was noted in 8 patients (8.2%) and resolved with conservative measures (Grade II) (Table 3).

**Table 3:** Postoperative complications classified by Clavien-Dindo grading and management approach (*n* = 97)

Complication	Number of Patients (*n*, %)	Clavien-Dindo Grade	Management Strategy
Pelvic collection	25 (25.8%)	II (*n* = 16), IIIa (*n* = 9)	Antibiotics (*n* = 16); image-guided drainage (*n* = 9)
Wound infection	15 (15.5%)	I–II	Dressing and antibiotics
Paralytic ileus	8 (8.2%)	II	Conservative (IV fluids, electrolyte correction)
Anastomotic leak	5 (5.2%)	II (*n* = 2), IIIb (*n* = 3)	Conservative (*n* = 2); diverting ileostomy (*n* = 3)
Urinary tract infection	5 (5.2%)	II	Antibiotics
Hydronephrosis	5 (5.2%)	IIIa (*n* = 3), II (*n* = 2)	Percutaneous nephrostomy (*n* = 3); observation (*n* = 2)
Perineal wound dehiscence	3 (3.1%)	II	Local wound care
Enterocutaneous fistula	3 (3.1%)	IIIb	Surgical repair
Stoma necrosis	3 (3.1%)	IIIb (*n* = 2), II (*n* = 1)	Stoma revision surgery (*n* = 2); wound care (*n* = 1)
Total Patients with ≥ 1 Complication	48 (49.5%)	–	–

• No Clavien-Dindo Grade IV (life-threatening) or Grade V (mortality) complications were reported

• Some patients experienced more than one complication; the highest-grade complication is reported per patient

• Grade I complications include any deviation from normal postoperative course not requiring intervention, generally included within wound infections managed conservatively

Other complications included anastomotic leak (*n* = 5; 5.2%), of which 3 were managed with diverting ileostomy (Grade IIIb) and 2 conservatively (Grade II); urinary tract infection (*n* = 5; Grade II); and hydronephrosis (*n* = 5), with 3 requiring percutaneous nephrostomy (Grade IIIa). Less frequent complications included perineal wound dehiscence(*n* = 3), managed with local wound care (Grade II), stoma necrosis (*n* = 3), with 2 requiring revision surgery (Grade IIIb), and enterocutaneous fistula (*n* = 3), all requiring surgical intervention (Grade IIIb). No Clavien-Dindo Grade IV or V (life-threatening or fatal) complications were recorded in this cohort.

The median follow-up was 25 months (1–117 months). Of 69 patients with R0 resection, 35 (50.7%) had recurrence, occurring more in LRRC versus LARC group (93.3% vs. 38.9%, *p* = 0.042).Among the 69 patients who achieved R0 resection, 26 patients (37.7%) underwent Total Pelvic Exenteration (TPE), and 43 patients (62.3%) underwent Modified Pelvic Exenteration (MPE). In our study, 28 out of 97 patients (28.9%) underwent non-R0 resection (R1/R2). 17 (60.7%) underwent Total Pelvic Exenteration (TPE), accounting for 71.9% of all TPE cases and 11 (39.3%) underwent Modified Pelvic Exenteration (MPE), representing 28.1% of all MPE cases (Table 4). Among the 15 patients with LRRC who underwent R0 resection, 11 patients (73.3%) underwent Total Pelvic Exenteration (TPE), 4 patients (26.7%) underwent Modified Pelvic Exenteration (MPE). Despite R0 resection, 14 of these 15 patients (93.3%) experienced recurrence.

**Table 4:** Recurrence amongst patients undergoing pelvic exenteration

Variable	LARC (*n* = 54)	LRRC (*n* = 15)	*P*-value
Recurrence after R0 resection			
Local alone	3 (5.6%)	4 (26.7%)	
Distant alone	9 (16.7%)	3 (20.0%)	
Local + distant	9 (16.7%)	7 (46.7%)	
Total recurrence	21 (38.9%)	14 (93.3%)	0.042

Local recurrence was noted in 4 LRRC patients (26.7%) compared to 3 LARC patients (5.6%). Distant recurrence occurred in 3 LRRC patients (20.0%) and 9 LARC patients (16.7%). Both local and distant recurrence were higher in LRRC (46.7%) versus LARC (16.7%), showing greater recurrence even after R0 resection. Recurrence amongst patients undergoing Pelvic Exenteration in shown in Table 4.

**Fig. 1:**
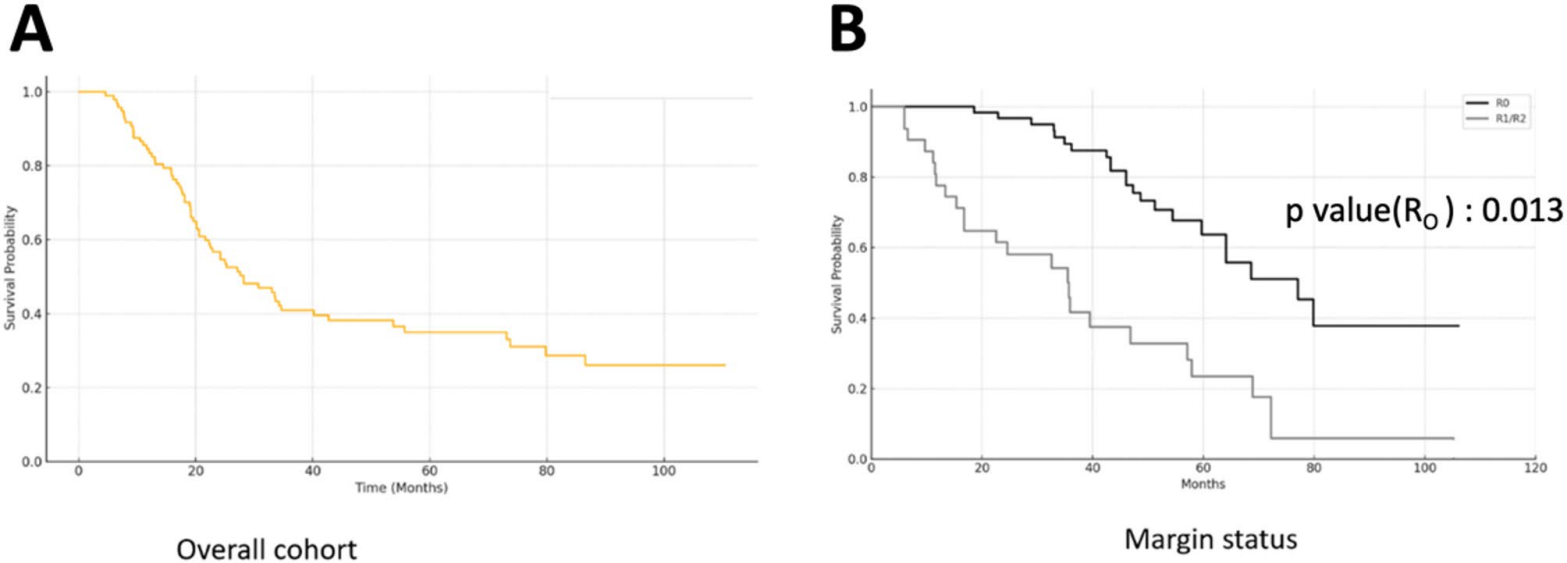
Kaplan–meier curve for overall survival in patients undergoing PE. Figure1**A**. Kaplan–meier curve for overall survival in the entire cohort Figure1**B**. Kaplan–meier curves comparing overall survival based on resection margin status

Univariate survival analysis in Table 5A was performed on all 97 patients, using Kaplan-Meier estimates that account for censored and uncensored data to provide time-point survival estimates. The 5-year OS values in Table 5A are derived from Kaplan-Meier curves, not from actual 5-year follow-up counts. The median follow-up was 25 months. Based on the Kaplan-Meier survival curve (Fig. 1A), approximately 39 patients (∼40%) were alive at 3 years and 26 patients (∼27%) at 5 years, reflecting survival estimates rather than actual follow-up counts. A significant difference in 5-year OS was observed between patients who achieved R0 resection and those with R1/R2 resections (51.9% vs. 12.9%, *p* = 0.013) (Fig. 1B). The median survival duration was markedly longer in patients undergoing PE for LARC, at 72 months, compared to 11 months in those treated for LRRC.

Tumor type and resection margin status significantly affected 5-year overall survival. LARC patients showed higher 5-year OS than LRRC patients (57.0% vs. 10.6%, *p* = 0.032). R0 resection yielded better survival than R1/R2 (51.9% vs. 12.9%, *p* = 0.013). On multivariate analysis using a Cox proportional hazards model, both primary disease presentation and R0 resection emerged as independent predictors of improved OS. Age, sex, tumor site, CEA level, and operation type were not associated with survival, as shown in Table 5.

**Table 5:** A. Univariate analysis of factors affecting 5-Year overall survival

Variable	*n*	5-year OS (%)	*p*-value
Age < 60 years	51	39.1%	0.158
Age ≥ 60 years	46	30.9%	–
Male	78	44.1%	0.415
Female	19	19.6%	–
Middle/Lower Rectum	65	30.1%	0.806
Upper Rectum/Rectosigmoid	32	46.7%	–
CEA < 5 ng/mL	38	26.2%	0.610
CEA ≥ 5 ng/mL	59	43.8%	–
LARC	65	57.0%	0.032
LRRC	32	10.6%	–
Total Pelvic Exenteration	43	24.3%	0.412
Modified Pelvic Exenteration	54	48.1%	–
R0 Resection	69	51.9%	0.013
R0 Resection: Total Pelvic Exenteration (TPE)	26		
R0 Resection: Modified Pelvic Exenteration (MPE)	43		
R1/R2 Resection	28	12.9%	–
Non-R0 Resection: Total Pelvic Exenteration (TPE)	17		
Non-R0 Resection: Modified Pelvic Exenteration (MPE)	11		

## Discussion

PE remains a cornerstone in the surgical management of LARC and LRRC, particularly when negative margins are achieved with limited data from Low and middle-income group countries, emphasizing our data as one of the largest single-institution experiences in LMICs, providing insights into the feasibility, outcomes, and challenges of complex oncologic surgeries in resource-constrained environments. Its uniqueness lies in a large cohort and extended follow-up in an LMIC setting, where pelvic exenteration data are limited due to systemic constraints Table 6.

**Table 6:** B. Multivariate cox regression analysis for predictors of 5-year overall survival

Variable	Hazard Ratio (95% CI)	*p*-value
Tumor Type (LARC vs. LRRC)	0.328 (0.141–0.763)	0.010
Resection Status (R0 vs. R1/R2)	0.397 (0.174–0.802)	0.033

This study of 97 patients over 20 years underscores key prognostic determinants and provides insights consistent with and additive to growing international literature on oncologic and perioperative outcomes of PE.

The male predominance in our series reflects the gender distribution of rectal cancer in India, where incidence is higher among men. Sociocultural factors and health-seeking behavior differences may contribute to earlier presentation in men.

Our study highlights that patients diagnosed with LARC have considerably better prospects regarding both recurrence and survival rates compared to those with LRRC. The 5-year OS rate in LARC patients (57.0%) was substantially higher than in LRRC (10.6%, *p* = 0.032), in line with recent findings from the International PelvEx Collaborative, which reported a 5-year OS of 52% in primary and 23% in recurrent disease [15, 16].

Postoperative morbidity following pelvic exenteration remains considerable, consistent with the complexity of the procedure. In our study, 49.5% of patients experienced at least one postoperative complication, most of which were Clavien-Dindo Grade II or lower, highlighting that many complications were manageable without major surgical intervention. Notably, pelvic collections and wound infections were the most frequent, and the majority were effectively treated with antibiotics or percutaneous drainage. More severe complications, such as anastomotic leaks and enterocutaneous fistulae, required reoperation or surgical diversion, falling under Grade IIIb morbidity. Despite the relatively high rate of complications, no Grade IV or V events were observed, reflecting the careful patient selection and perioperative management strategies implemented at our center.

The grading of morbidity using Clavien-Dindo provides transparency and facilitates comparison with other large PE series. It also underscores the need for multidisciplinary perioperative planning, and in future, incorporating Enhanced Recovery After Surgery (ERAS) protocols may help reduce postoperative complications and improve recovery.

In our multivariate analysis, achieving an R0 resection was identified as the most important prognostic factor (HR: 0.397; *p* = 0.033). This finding is consistent with the research by Radwan et al. and recent meta-analyses, which emphasize that R0 resection is the key predictor of long-term survival in both initial and recurrent cases [17, 18].

All patients underwent standard preoperative imaging with pelvic MRI and/or PET-CT. However, 28.9% had R1/R2 resections, mainly due to underestimated tumor extent, especially in recurrent or post-radiotherapy cases where fibrosis and distorted anatomy limit radiologic accuracy. Intraoperative findings revealed occult invasion of critical structures not seen preoperatively. In select cases, non-R0 resection was chosen, balancing oncologic goals with acceptable morbidity.

Our findings indicated that patients with LRRC experienced high rates of recurrence even after undergoing R0 resection, with a rate of 93.3% compared to 38.9% in those with LARC. The most frequent type of recurrence involved both local and distant sites. This pattern of recurrence could be due to the microscopic spread of the disease beyond what is visible on radiological scans, coupled with the limited ability to perform wider excisions because of anatomical limitations [19]. Among the 15 patients with locally recurrent rectal cancer (LRRC) who underwent R0 resection, 11 (73.3%) underwent Total Pelvic Exenteration (TPE), while 4 (26.7%) underwent Modified Pelvic Exenteration (MPE). Despite achieving macroscopically complete (R0) resection, 14 of these 15 patients (93.3%) developed further recurrence. This exceptionally high recurrence rate underscores the aggressive tumor biology of LRRC and the challenges in achieving durable disease control, even with radical surgical interventions in previously treated patients, although detailed molecular or genomic characterization was not available in this cohort.

Molecular studies have started dissecting transcriptomic differences between primary and recurrent rectal tumors. A study by Wang et al. found overexpression of EMT-related and immune-evasive genes in LRRC, potentially causing higher therapy resistance and aggressive recurrence patterns [20]. 

MPE was performed more often in LARC patients (69.2%) and showed reduced operative time, fewer transfusions, and shorter hospital stays versus TPE. Das et al. reported similar oncological outcomes in MPE versus TPE when negative margins were achieved [21]. 

Despite worse surgical outcomes in LRRC, pathological features like tumor differentiation, pT stage, nodal status, LVI, and PNI were comparable between groups. The trend towards using tumor regression grade (TRG), immune infiltrate density, and ctDNA dynamics post-CRT may offer predictive value for stratification [22, 23].

Limitations of this study include its retrospective design, single-institution setting, and inherent selection bias. Quality-of-life (QoL) assessments were not routinely recorded, which is increasingly recognized as a critical endpoint in PE literature [24]. We acknowledge that MPE involves tailored resections based on disease extent and potential organ preservation, including bladder preservation in posterior exenteration cases, vaginal preservation when the anterior wall was spared, and urethral or anal sphincter preservation in selected low-volume central recurrences. However, detailed documentation of these modifications was limited during the study period, preventing formal analysis of their impact on margins and functional outcomes; we plan to implement a detailed organ-specific classification in future data collection.

Another important limitation was the variable access to advanced imaging over the 20 years. While contrast-enhanced CT and MRI were routinely used, high-resolution pelvic MRI and PET-CT were not consistently available, particularly in the early years, and many referred patients had incomplete or unavailable prior imaging. Resource and cost constraints sometimes limited repeated or advanced imaging, necessitating reliance on clinical assessment and intraoperative findings. These factors underscore the challenges of optimal preoperative staging, particularly in LRRC cases, and highlight the critical role of multidisciplinary decision-making in tailoring surgical approaches and achieving the best possible oncologic outcomes.

Despite these limitations, study strengths include a large sample size and extended follow-up. This study fills a critical void in oncologic literature from low- and middle-income countries (LMICs), where exenteration data is scarce due to resource and logistic constraints.

Future research should focus on prospective multicenter studies to systematically evaluate patient-reported outcomes, functional recovery, and value-based endpoints, which are increasingly recognized as critical measures of success in pelvic exenteration. To further improve R0 resection rates, strategies should include meticulous preoperative planning using high-resolution imaging, multidisciplinary evaluation, neoadjuvant therapy for tumor downstaging, and tailored organ-preserving surgical approaches. Intraoperative margin assessment and careful surgical technique are also essential to optimize complete resection while balancing oncologic radicality with postoperative function and quality of life.

## Conclusion

Pelvic exenteration (PE) offers a potentially curative option for selected patients with locally advanced rectal cancer (LARC) and locally recurrent rectal cancer (LRRC), with our 20-year single-institution experience demonstrating significantly better outcomes in LARC than LRRC. R0 resection was the most important independent predictor of overall survival, underscoring the critical need for meticulous preoperative planning and multidisciplinary evaluation. While PE remains associated with considerable morbidity, especially in LRRC cases, modified and tailored surgical approaches can improve feasibility and patient outcomes. Despite achieving clear margins, LRRC cases had higher recurrence and poorer survival, likely reflecting underlying tumor biology and anatomical constraints. Our study contributes important real-world data from a high-volume tertiary center in a low- and middle-income country, where evidence on PE remains scarce. Future efforts should focus on improved patient selection through integration of molecular, imaging, and functional data to enhance oncologic outcomes while preserving quality of life.

## Data Availability

The datasets used and/or analysed during the current study are available from the corresponding author on reasonable request.

## Abbreviations

PE: Pelvic Exenteration
LARC: Locally Advanced Rectal Cancer
LRRC: Locally Recurrent Rectal Cancer
TPE: Total Pelvic Exenteration
MPE: Modified Pelvic Exenteration
OS: Overall Survival
R0 resection: Microscopic margin-negative resection
R1 resection: Microscopic margin-positive resection
R2 resection: Macroscopic residual tumor
CRT: Chemoradiotherapy
TNT: Total Neoadjuvant Therapy
TME: Total Mesorectal Excision
MDT: Multidisciplinary Team
CEA: Carcinoembryonic Antigen
CT: Computed Tomography
MRI: Magnetic Resonance Imaging
PET-CT: Positron Emission Tomography-Computed Tomography
LAR: Low Anterior Resection
HR: Hazard Ratio
CI: Confidence Interval
SPSS: Statistical Package for the Social Sciences
